# Discordância da Lipoproteína de Baixa Densidade e da Lipoproteína de Alta Densidade com Gravidade de Doença Arterial Coronariana

**DOI:** 10.36660/abc.20200033

**Published:** 2020-04-06

**Authors:** Iran Castro, Hugo Fontana

**Affiliations:** 1 Instituto de CardiologiaFundação Universitária de CardiologiaPorto AlegreRSBrasilInstituto de Cardiologia - Fundação Universitária de Cardiologia,Porto Alegre, RS - Brasil

**Keywords:** Doenças Cardiovasculares/mortalidade, Lipoproteinas LDL, Lipoproteinas HDL, Doença da Artéria Coronariana, Inibidores de Hidroximetilglutaril-CoA Redutases, Pro-Proteina Convertase 9

As doenças cardiovasculares (DCVs) são as principais causas de morte em todo o mundo.^1^ A dislipidemia é um fator de risco e também um fator causal de DCVs, sendo foco da terapia de prevenção primária e secundária das DCVs.

Existe um consenso e um amplo conhecimento acerca dos mecanismos causais da lipoproteína de baixa densidade (LDL) nas DCVs, e os benefícios da terapia hipolipemiante, sendo que a magnitude de seu efeito é proporcional à redução dos níveis circulantes.^2^ No entanto, apesar do uso intensivo de agentes hipolipemiantes, permanece um risco residual, um alvo constante de pesquisa e terapia.

Evidência recente confirma que o evento inicial da aterogênese é a retenção de LDL e outras partículas na parede do vaso.^3^ Níveis elevados de colesterol não-HDL ajudam a identificar pacientes que continuam com alto risco para eventos cardiovasculares apesar de níveis baixos de LDL.^4^

O presente estudo^5^ avaliou retrospectivamente características anatômicas de 574 pacientes diagnosticados com síndrome coronariana aguda e correlacionou os achados com níveis séricos de LDL e não-HDL. Observou-se uma discordância de 15% entre LDL e não-HDL, o que foi similar a estudos prévios.^6^ No entanto, não foram identificadas diferenças anatômicas significativas na avaliação da gravidade da doença aterosclerótica. O uso prévio de estatinas tem um efeito mais significativo na redução de LDL em comparação a não-HDL, o que pode explicar a falta de associação.

Além disso, a sensibilidade dos níveis de LDL em identificar risco cardiovascular é menor no diabetes.^7^ De fato, o presente estudo identificou discrepâncias na associação entre diabetes mellitus e níveis de LDL. Talvez o tamanho da amostra não tenha sido adequado para identificar diferenças anatômicas entre os grupos. O acompanhamento de pacientes com associações discrepantes poderia identificar um subgrupo de pacientes em alto risco para novos eventos cardiovasculares.

As diretrizes brasileiras já incluem metas de LDL e não-HDL, buscando identificar indivíduos com níveis adequados de LDL e alto risco residual para eventos cardiovasculares. Estudos prospectivos certamente ajudarão a identificar o subgrupo de pacientes que requeiram uma abordagem terapêutica mais intensa, e que provavelmente se beneficiariam de terapias mais caras e efetivas, como inibidores de PCSK9 e lomitapida.^8^ Estudos anteriores já mostraram a eficácia desses medicamentos na redução de eventos cardiovasculares e apolipoprotein(a) em alguns grupos de pacientes.
