# Supplementary material for: Discordância da Lipoproteína de Baixa Densidade e da Lipoproteína de Alta Densidade com Gravidade de Doença Arterial Coronariana
Source: Arq Bras Cardiol. 2020 Apr 6;114(3):476. [Article in Portuguese] doi: 10.36660/abc.20200033 (PMC7792721; doi:10.36660/abc.20200033)
Supplement: Translation

# Translation: Discordance of Low-Density Lipoprotein Cholesterol and Non-High-Density Lipoprotein Cholesterol with Severity of Coronary Artery Disease

**Authors:** Iran Castro, Hugo Fontana

**Affiliations:** 1 BrazilInstituto de Cardiologia - Fundação Universitária de Cardiologia, Porto Alegre, RS - Brazil

**Keywords:** Cardiovascular Diseases/mortality, Lipoproteins,LDL, lipoproteins,HDL, Coronary Artery Disease, Hyydroxymethylglutaryl-CoA Reductase Inhibitors, Proprotein Convertase 9

Cardiovascular diseases (CVDs) are the leading cause of mortality world.^1^ Dyslipidemia is a risk and causal factor and is the focus of the therapy for primary and secondary prevention of CVDs.

There is consensus and broad understanding of the causal mechanisms of low-density lipoproteins (LDL) in CVDs, and the benefit of the hypolipidemic therapy, with a magnitude of effect proportional to the reduction in serum levels.^2^ However, despite intensive use of lipid lowering agents, there remains a residual risk, a constant target of research and therapy.

Recent evidence confirms that the initial event of atherogenesis is the retention of LDL and other particles in the vessel wall.^3^ High non-HDL cholesterol levels help identify patients who despite having low serum LDL levels remain at high risk for cardiovascular events.^4^

The present study^5^ retrospectively evaluated the anatomical characteristics of 574 patients diagnosed with acute coronary syndrome and correlated the findings with LDL and non-HDL serum levels. There was 15% disagreement between LDL and non-HDL, which was similar to previous studies.^6^ However, no significant anatomical differences were identified in the assessment of the severity of atherosclerotic disease. Prior use of statins may have a more significant effect on LDL reduction than non-HDL, which may explain the lack of association.

In addition, the sensitivity of LDL levels in identifying cardiovascular risk is reduced in diabetes.^7^ In fact, the present study reported discrepancies in the association between diabetes mellitus and LDL levels. Perhaps the sample size was not adequate enough to evidence anatomical differences between the groups. The follow-up of patients with discrepant associations could identify a subgroup at higher risk of new events.

The Brazilian guideline already includes both LDL and non-HDL targets, seeking to identify individuals with high residual risk of cardiovascular events despite adequate LDL levels. Prospective studies will help better identify the subgroup of patients who require more intense treatment approach, who would probably benefit from more expensive and effective therapies such as PCSK9 inhibitor and Lomitapide.^8^ These drugs have already been shown to be effective in reducing cardiovascular events and apolipoprotein(a) in some groups of patients.
